# Digital Phenotyping: Data-Driven Psychiatry to Redefine Mental Health

**DOI:** 10.2196/44502

**Published:** 2023-10-04

**Authors:** Antoine Oudin, Redwan Maatoug, Alexis Bourla, Florian Ferreri, Olivier Bonnot, Bruno Millet, Félix Schoeller, Stéphane Mouchabac, Vladimir Adrien

**Affiliations:** 1 Infrastructure for Clinical Research in Neurosciences Paris Brain Institute Sorbonne University- Institut national de la santé et de la recherche médicale - Centre national de la recherche scientifique Paris France; 2 Department of Psychiatry Pitié-Salpêtrière Hospital Public Hospitals of Sorbonne University Paris France; 3 Department of Psychiatry Saint-Antoine Hospital Public Hospitals of Sorbonne University Paris France; 4 Medical Strategy and Innovation Department, Clariane Paris France; 5 NeuroStim Psychiatry Practice Paris France; 6 Department of Child and Adolescent Psychiatry Nantes University Hospital Nantes France; 7 Pays de la Loire Psychology Laboratory Nantes University Nantes France; 8 Institute for Advanced Consciousness Studies Santa Monica, CA United States; 9 Media Lab Massachusetts Institute of Technology Cambridge, MA United States

**Keywords:** digital phenotype, empowerment, mental health, personalized medicine, psychiatry

## Abstract

The term “digital phenotype” refers to the digital footprint left by patient-environment interactions. It has potential for both research and clinical applications but challenges our conception of health care by opposing 2 distinct approaches to medicine: one centered on illness with the aim of classifying and curing disease, and the other centered on patients, their personal distress, and their lived experiences. In the context of mental health and psychiatry, the potential benefits of digital phenotyping include creating new avenues for treatment and enabling patients to take control of their own well-being. However, this comes at the cost of sacrificing the fundamental human element of psychotherapy, which is crucial to addressing patients’ distress. In this viewpoint paper, we discuss the advances rendered possible by digital phenotyping and highlight the risk that this technology may pose by partially excluding health care professionals from the diagnosis and therapeutic process, thereby foregoing an essential dimension of care. We conclude by setting out concrete recommendations on how to improve current digital phenotyping technology so that it can be harnessed to redefine mental health by empowering patients without alienating them.

## Introduction

The emergence and rapid adoption of digital technologies in medicine have led to changes in medical practice and the conception of health. One such technology is based on the notion of the digital phenotype, which first emerged in 2015 in *Nature Biotechnology* [1], building on Dawkins’ extended phenotype, defined as the set of observable characteristics or traits of an organism. Digital phenotyping (DP) refers to the collection of observable and measurable characteristics, traits, or behaviors of an individual, defined as “moment-by-moment quantification of the individual-level human phenotype in situ using data from personal digital devices” [2]. The data can be divided into active and passive subgroups. Active data requires user engagement (eg, the completion of a questionnaire), while passive data is collected without user participation or notification.

The use of passive data collection through sensors of all kinds represents a step change in the clinical observation of patients, as it gathers fine-grained information that can be more relevant to illness phenotypes than the exclusively active data collection by the patient (eg, ecological momentary assessment and chatbot interactions). Today, there is a proliferation of digital interfaces as each individual interacts with a variety of connected objects, including wearables and smartphones equipped with a plethora of measurement tools. They can store and measure different types of data, including GPS data, proximity to other devices using Bluetooth, walking speed using an accelerometer, heart rate, oxygen level, electrical cardiac activity, sleep quality, perspiration using humidity sensors, tone of voice, activity on social networks, the lexical field of written sentences, etc. The collection of passive data has already led to some progress in various medical disciplines (eg, for monitoring cognitive function in cognitive impairments [3], Parkinson’s progression [4], cardiac electrophysiology [5], seizure detection [6], and glucose in diabetes [7]. DP serves the dual purpose of fulfilling clinical objectives and logistical aims. The clinical goals include improving health care professionals’ (HCPs’) ability to diagnose patients and select the most effective treatment options. Meanwhile, the logistical objectives involve managing health care systems to ensure optimal performance and efficiency. Nevertheless, DP may also constrain the role of HCPs, who already rely on clinical decision support systems, structuring the profession in a top-down manner at the risk of subjugating and disqualifying their know-how. Furthermore, the collection of quantitative data may dispossess patients of their subjective distress [8].

We believe that the emergence of technologies such as DP in medicine underscores the fundamental differences between 2 complementary conceptions of health [9]: one centered on the illness, the other on the patient. Illness-centered medicine has its roots in the ancient Greek medical school of Knidos. The aim is to cure illnesses. It is a medicine focused on the diagnosis and classification of illnesses. It can be related to the myth of Prometheus (ie, delaying or denying death) and corresponds to the objectification of the patient as a body or machine made up of organs and functional systems. Treatments involve invasive gestures (punctures, incisions, etc), the requirement to take medication, or, in the field of psychiatry, neurostimulation techniques such as electroconvulsive therapy. Behind this aggressive dimension of care [10] lies the idea of combating nature rather than seeking to improve coexistence with it. Patient-centered medicine is derived from the Hippocratic tradition. The aim is to care for patients by focusing on their self-experience of their illness, just as in palliative care, where human interactions are fundamental. This holistic (ie, whole person) approach to medicine focuses on prognosis and involves considering mental and social factors in order to improve individual patients’ quality of life. Throughout history, the conception of health has been pulled in these 2 opposite directions, depending on the patient’s or HCP’s point of view [10]. In the past decade, there has been much progress in patient-centered medicine. For example, clinical trials are increasingly using patient-reported outcome measures, as these are now being demanded by health authorities and regulatory agencies [11].

This paper examines how the implementation of DP in psychiatry could redefine mental health. This will be a challenging process owing to the plurality of concepts and approaches it involves. The World Health Organization defines health as a “state of complete physical, mental, and social well-being and not merely the absence of disease or infirmity” [12] and applies a normative approach based on a set of arbitrary conditions. Psychiatry has always claimed to be clinical medicine. In contrast to other medical disciplines, it has no consensual biological markers and no gold standard to help HCPs (ie, psychiatrists, psychologists, nurses, social workers, and therapists) establish diagnosis. The criteria used in psychiatry are clinical and mostly qualitative, stemming from observations of bedridden patients, in accordance with the etymology of this ancestral term (klinê, meaning bed). HCPs must make a thorough assessment of the functional impairment caused by the psychiatric illness in terms of the individual-environment interaction to justify the treatment. Finally, what makes psychiatry so complex is that there are no standards either for the physiopathological explanation of illnesses or for therapeutics. There is no international consensus on therapeutic guidelines, and there is huge interindividual variability in treatment response and tolerance, whether that treatment is pharmacological or psychotherapeutic. In other words, what is beneficial for one patient may not be for another. The same applies to physiopathological models, which range from psychodynamics to neurobiology and from genetic predispositions to environmental factors. In psychiatry, being in poor health can even have secondary benefits [13].

Classifications have been drawn up to justify therapeutic interventions. These can be either categorical, such as the Diagnostic and Statistical Manual of Mental Disorders, Fifth Edition (DSM-5) [14] and the International Classification of Diseases, or dimensional, such as the “research domain criteria” (RDoC) [15]. Both conceptions have issues [16]. Categorical classification reflects medical tradition but leaves some help-seekers without care due to arbitrary diagnostic thresholds. As they have low intrinsic validity, categorical illnesses also give rise to frontier forms and disorder spectrums with little temporal validity or therapeutic interest [17,18]. Dimensional classification considers all dimensions to be equal in their pathogenicity without taking account of their interplay and causal links (eg, some dimensions may be defense mechanisms from an evolutionary or psychodynamic perspective). It also requires thresholds that are well defined, given that they are exposed to HCPs’ subjectivity. Finally, some dimensions are purely descriptive and do not consider functional impairment or therapeutic implications [19,20].

To delve into these matters, in this viewpoint paper, we begin by discussing the expectations related to the rise of a data-driven approach to mental health in the form of DP. Subsequently, we examine how its use threatens to dehumanize mental health. Lastly, we set out guidelines for ensuring that the implementation of DP in psychiatry fosters more patient-centered mental health.

## Toward a Patient-Centered Mental Health

### Emergence of Digital Phenotyping in Psychiatry

Data science emerged in psychiatry some years ago, representing all the digital information about mental health, individuals’ properties, and digital factors involved in the health care processes. It ranges from theoretical variables of interest such as major life events, comorbid diagnosis, and stress factors to blood marker levels and clinical characteristics to functional neuroimaging [21]. DP gives HCPs a new set of digital biomarkers, collected from wearables, smartphones, but also virtual reality devices [22] or in gaming contexts [23], offering the opportunity to model mental health [24] and the extent of individual-environment interactions.

**Figure 1:**
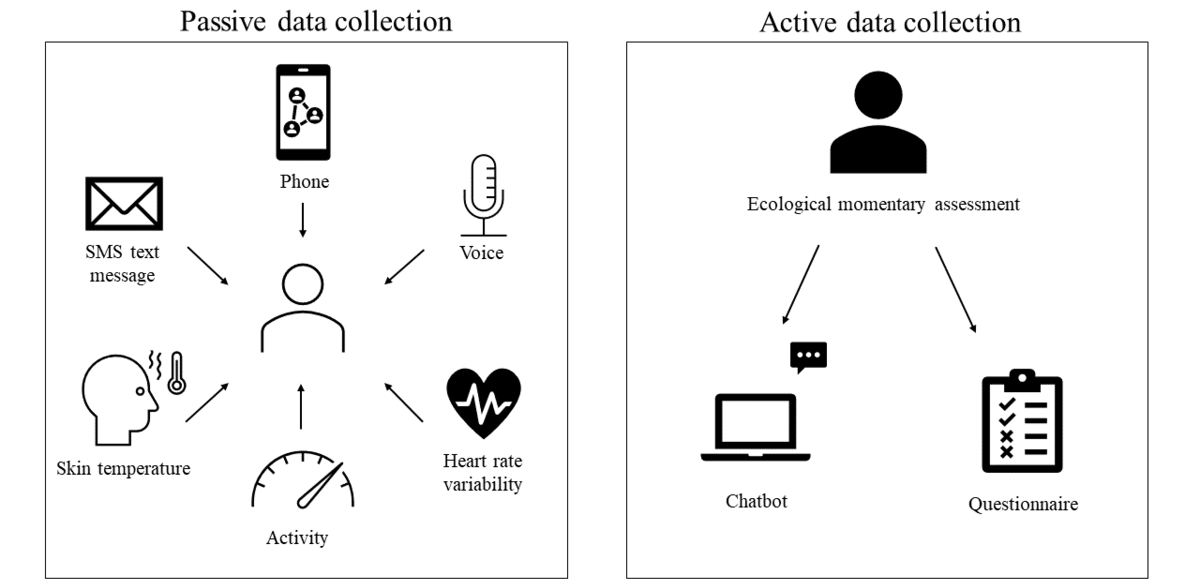
Schematic representation of digital phenotyping.

Machine learning brings powerful tools to explore high-dimensional and real-time data concerned with DP. It offers the opportunity “to make sense” of these digital signs of the reality they try to represent in the state of mental health [25-27]. Some see in this technology the potential to better understand the neurobiological mechanisms underlying psychiatric illnesses [28] or give new transdiagnostic models of comprehension of symptoms, in line with the “RDoC” perspective [29]. In addition, ML could be used to deliver new predictive models. Artificial intelligence (AI) might be capable of “overcoming the trial and error-driven status quo in mental health care by supporting precise diagnoses, prognosis, and therapeutic choices” [30]. These systems may have the ability to predict risks, help HCPs make clinical decisions, increase the accuracy and speed of diagnosis, and facilitate the examination of health records. Integrating this data-driven approach into clinical practice could reduce the workload of HCPs. They can already efficiently perform tasks such as diagnosing skin diseases and analyzing medical images (eg, in neurology, ophthalmology, cardiology, and gastro-enterology) [31,32] and could soon be used in clinical decision support systems in psychiatry [33], making compulsory admissions more helpful [34]. DP therefore has a natural home in psychiatry [35,36], helping HCPs access a new set of data based on individuals’ behavioral experiences and increasing their ability to classify and understand symptoms in their contextual and temporal dimensions, as well as the illnesses themselves [37,38]. Data reflecting emotions, levels of energy, behavioral changes [39], symptoms such as sociability, mood, physical activity, and sleep [40], but also logorrhea, agitation, rumination, hallucinations, or suicidal thoughts, could constitute the digital signature of a pathology (Figure 1). Given that psychiatry usually explains a mental illness and its issues in terms of the difficulty patients have interacting with their environment, it is only logical for DP to arouse such interest, especially as it could compensate for the absence of reliable biomarkers.

### An Improved Psychiatric Care

In recent decades, the psychometric assessment of patients has involved self-report questionnaires (eg, the Patient Health Questionnaire for Depression [41]) and observer-rated scales (eg, the Hamilton Rating Scale for Depression [42]). As these scales are filled in either by the patient or the HCP, they necessarily have a degree of subjectivity. DP would enable phenomenological data to be collected, with the possibility of establishing ecologically valid psychometric assessments [37,43] (Figure 1). For instance, user-generated content on social media sites such as Reddit allows for the recognition of mental illness-related posts with good accuracy using a deep learning approach [44].

Various studies have already provided evidence about the use of DP for diagnostic purposes. As an example, there is a correlation between circadian rhythm, step counts, or heart rate variability and the diagnosis of a mood disorder or mood episode [45-48]. Other correlations have been found between data and symptoms of schizophrenia [49-51], major depression [52-57], mood disorders [46,58,59], posttraumatic stress disorder [60,61], generalized anxiety disorder [62], suicidal thoughts [63,64], sleep disorders [65], addiction [66], stress [53,67], postpartum [68,69], autism [70], and child and adolescent psychiatry [71,72]. Among other examples of the efficiency of DP for prediction or diagnosis in mental health, Instagram photos or Facebook language have been found to be predictors of depression [73,74]; suicidal risk could be assessed from social media [75,76] with increasing precision if DP would integrate electronic health records data [77,78]; automated analysis of free speech can measure relevant mental health changes in emergent psychosis [79] or incoherence in speech in schizophrenia [80]. It is worth noting that clinical utility may be derived from a combination of passive and active data and not from passive data alone [81]. In total, increasing diagnostic accuracy could help avoid treatment delays or errors. For example, there is currently an average 8-year delay in the diagnosis of bipolar illness in France [82]. Concerning the choice of medication, phenotypic markers already play a major role (eg, antidepressants for depression, whether it is accompanied by insomnia or hypersomnia). DP could bring together more phenotypic markers than HCPs can collect and thus improve the choice of treatment, countering the cognitive bias effect on decision-making [83].

Finally, DP could improve the follow-up of people with serious mental illnesses and optimize care between 2 consultations, especially when accessing care facilities can be difficult. It could also improve the assessment of treatment efficiency. Some researchers have hypothesized that heart rate variability is influenced by the progression of the patient’s depression and could be a new biomarker of treatment response [84,85]. High diagnostic confidence improves treatment compliance, and problems with compliance are a frequent source of relapse in mental illness [86]. DP could in turn provide useful tools for enhancing therapeutic education and improving the prediction of relapses [48,87-90], Ultimately, DP could also be used to assess whether patients could benefit from psychotherapy [91], using parameters with proven efficiency [45], as part of an evidence-based medicine strategy.

### Patient’s Empowerment

DP introduces new capacities to assess differences between what is normal and what is pathological. Canguilhem [92] identified normative capacity (ie, normativity) as a central condition for gauging to what extent a living person is in good health: “What characterizes health is the possibility to tolerate infractions of the usual norm and to set new norms for new situations.” Thus, all living beings live with their own norms based on their specific biological limits. These norms are defined by Canguilhem [92] as a mode of functioning in certain environmental conditions that allows individuals to have normal abilities and live normal lives. Unlike machines, living beings have the possibility of defining their norms according to environmental conditions in order to ensure real-time adaptation. The limits of these norms are inevitably tested in the course of the individuals’ biological lives as they interact with their environment and pursue their life goals, and when they have a disability that prevents them from meeting their goals, they feel ill. Nonetheless, this experience of the limits is based on self-feeling and is totally personal. When this normative capacity no longer allows an individual to adapt, thus triggering an illness, it is legitimate to intervene in order to restore the ability to set new life goals. Concerning psychiatric symptoms, DP could add a digital dimension to the notion of limits and the production and assessment of norms. Experiencing physical pain when one’s leg is broken is more readily acknowledged, and seeking help is a common response. However, people often encounter challenges in recognizing psychic pain [93], depressive symptoms, or anxiety and find it much more difficult to perceive these conditions as abnormal and deserving of appropriate adaptations or treatment. DP could provide a novel and sensitive approach that empowers individuals to define their own digital mental health and establish their own digital norms. In accordance with Canguilhem’s principles, the threshold between the normal and the pathological would become more nuanced [92,94]. Individuals would be able to perceive mental health as something more tangible, allowing them to determine for themselves which conditions contribute to their well-being independently of any scientific paradigms used by HCPs for their evaluation and treatment approaches. Consequently, individuals would have the opportunity to take charge of their mental health care, paving the way for a comprehensive mental health prevention system. With this perspective, it would be simpler to harness individual motivation for making changes and foster active participation in health strategies. In the United States, data-driven psychiatry is starting to emerge, with a close relationship among patients, HCPs, and DP [95]. There have been attempts at self-management, where patients are given greater autonomy with regard to technologies and the management of their symptoms [94]. For instance, some open science applications such as mindLAMP (which collects health data, produces easy-to-understand graphs, enables journaling thoughts and reflections, and offers customized mental health interventions) allow some depressed or alcoholic patients to rapidly develop emotional self-awareness, track thinking patterns in real time, gain insight into their progress, connect to the clinical team, engage with medication, or reinforce their confidence in psychotherapy [96]. Other studies have suggested that DP increases patients’ feelings of control over their symptoms [97]. The quantified self-approach could help scientists understand pathology better and deserves further exploration. In Western medicine, representations of diseases and health are biased by cultural interpretation. Thanks to the new language through the data concerning the self-evaluation close to a data-feeling, patients could free themselves from this bias. Herein lies the idea of compensating for the subjectivity of clinical interviews. DP could introduce a more precise and decisive point of view of the daily life of the patient, where functional impairments, one of the major characteristics of mental illness, must not be neglected. DP is therefore a potential source of objectivity in mental illnesses [98], making it possible to flag up daily abnormalities.

### Toward a Personalized Psychiatry

The possibility of empowerment is consistent with psychiatry’s move toward a more personalized approach. It was initially conceived of as the tailoring of psychiatric practices to the patient’s situation based on the HCP’s assessment. However, it could take the form of personalized requests from patients to HCPs, with better comprehension of their mental disorders converting patients into self-researchers investigating their own illness. DP could create a unique network of macro (social and smart cities), meso (relation to environment and situations), micro (the person), and very micro (physiological mechanisms) data integrated into the patient’s daily life. DP would promote personalized psychiatry [99], doing away with the usual lengthy periods of observation in psychiatry. This corresponds to the concept of intelligent health, defined as the expansion of electronic health through the inclusion of built-in data analysis using new technologies, as well as the extension of patient assessment to the patient’s environment and HCPs, and data mining to support decision-making [100]. HCPs and patients must be able to select the parameters that match their characteristics and situation in a move toward clinical augmentation. This added precision in clinical observation should lead to equally shared (between HCPs and patients) models of illnesses and treatments. It opens the way to patient-centered mental health, where “the symptom network” paradigm takes its place for a better phenotypic characterization of disorders and their evolution. The symptom network aims at a technologically augmented clinical and therapeutic relationship [101] that could thus explore how symptoms influence each other without trying to find a unique causal source in a more consistent and transparent psychopathological framework [102].

To conclude, we can see how DP could bring about major scientific progress in psychiatry toward patient-centered mental health. Nevertheless, this optimistic view of its potential uses or advantages needs to be tempered by practical issues. This quest for optimized performance could also have negative impacts (Textbox 1) on society that still need to be assessed.

**Positive and negative impacts of mental health as defined by digital phenotyping on society.**

**Positive impacts of patient-centered mental health**

- Precise, continuous, multidimensional psychometric assessment.
- Deeper and more precise understanding of mental illnesses.
- Faster and more accurate diagnosis of mental illnesses.
- Better-adapted treatment based on each patient’s specificities, life history, and needs.
- Better follow-up and prognosis of the mental illness over time and in changing conditions (eg, outside the hospital).
- Patients are empowered to monitor the progression and root causes of their disease.
- All in all: a precise, personalized (in the technical sense), patient-centered definition of mental health.

**Negative impacts of dehumanized mental health**

- Nominalism and arbitrariness. It is unclear who or what decides the norms and standards beyond mere statistical means. Norms are disconnected from any lived experience.
- Reproducing bias and systematic error in the understanding of mental illnesses: only relative objectivity.
- Depriving patients of their agency, self-perception, and personal definition of what it means to be healthy and well.
- Creation of arbitrary standards with a high risk of normativity, generating guilt and self-image issues.
- Biopower: surveillance and privacy issues.
- Alienated patients experience life in the second person after the technology and the disease.
- All in all: decentering of the human being, disappearance of the human dimension of mental health.

## Risk of Dehumanizing Mental Health

### Overview

Although DP introduces the possibility of individuals managing their own health data, increasing the amount of information available for them to assess their own health status, there might also be a process of reduction or simplification that would have massive consequences for patients’ self-determination. Moreover, as their health data would be more readily available to third parties, it might end up being used for other purposes besides improving their health.

### Concerns With the Implementation of Digital Phenotyping in Psychiatry

The clinical interview is the moment when patients’ complaints are heard by HCPs and their distress is recognized. Their illness is discussed and diagnosed, and an appropriate treatment is prescribed. Subjective representations of individuals and HCPs are part of the decision-making system in psychiatry, shaping discussions about the individual's state of health and illness. In striving to improve the current state of health, a shared objective guides the HCPs’ decisions. However, considering the data yielded by DP analysis, the decision-making process in psychiatry might undergo profound transformations to align with the evolving understanding of mental health. Canguilhem [92] explained that the risk of nominalism is to reduce life to machine functioning. For example, a healthy individual may start to feel ill after seeing figures that deviate from the norm, while a help-seeker may be declared healthy by DP. The patient’s status may thus become disconnected from the felt experience [103], excluding self-construal, illness narratives, interpersonal dynamics, and social contexts, which are determinants of mental health [18]. The term data-driven (in society, marketing, health, etc) already exists and is used when decision-making is mainly based on data interpretation. The question of who creates the norms and indicators does not seem to be an issue at present. By default, these conditions are fixed either by researchers on the basis of their study findings or by the designers of health applications (eg, walking 10,000 steps per day, limiting screen time to 4 hours per day, and eating certain types of food selected by an algorithm). For example, a marketing campaign by Yamasa Corporation claimed that walking 10,000 steps per day is a factor for well-being. However, the goal was to promote a step tracker during the 1964 Tokyo Olympics. They called this technology manpo-kei, which in Japanese literally means “10,000 steps counter.” A systematic review acknowledged the association between walking and the reduction of all causes of mortality [104] but concluded that we can expect more benefits with every 1000 steps we walk beyond 8000. The 10,000-step target is therefore arbitrary and should be reconsidered. This example helps us understand that many recent applications continue to rely on approximations of scientific data [105,106]. At present, too many applications are dedicated to different types of data collection and analysis methods that are not well assimilated [40,107]. Using norms set by DP could deprive individuals of the possibility of discussing which norms they should strive toward in order to achieve better well-being. Studies on DP in psychiatry have limited research interest [108], none of which has been applied to clinical diagnosis and treatment or shown improvements in mental health over the long term. Some applications, such as Instagram, are already suspected of damaging users’ mental health [109].

Froment [103] pointed out that historically speaking, the term illness is not technical but undeniably profane and phenomenological. The science of medicine was built on a prescientific conception of illnesses but has gradually adopted concepts developed by HCPs and health authorities. If DP modifies these concepts, it could compromise patients’ quest to understand their mental health as well as the aim of the treatment. Beyond the very definition of mental health, there is an issue with the power of data over people’s freedom and way of life in society. It compromises the very possibility of innovative objectivity or may lead to the outright failure of any attempt to achieve objectivity through this technology.

### Relative Objectivity

Datafication, which is defined as the tendency to overrepresent objects with digital data (eg, DP in psychiatry), has several detractors, especially when it comes to the possibility of data representing part of the real world [93,110-112]. For instance, several scales have been developed to quantify psychic pain, but they are very heterogeneous in terms of content, and cannot be compared or replaced by each other [112]. Data power may therefore paradoxically deplete reality [113]. Each of the many steps between data collection, analysis by an algorithm, and implementation by patients or HCPs is a source of bias [114-119]. Algorithmic bias emerges when databases are created without exhaustive data or when data is sourced from patients with coexisting illnesses [120,121]. Additionally, machine learning algorithms give rise to the black box effect (ie, the opacity of the internal processes of a system that produce outputs from inputs) [122]. This concern appears when users (eg, HCPs or patients) lack knowledge about the inner workings of the system, rendering them unable to explain the outcomes of an analysis [123]. Studies exploring AI-based diagnosis do not take either difficult cases or HCPs’ clinical experience into account. AI makes mistakes when HCPs perform poorly [124]. Machine learning searches for statistical invariants, whereas clinical experience provides tacit data (ie, heuristics coupled with models or concepts, used unconsciously by HCPs, and which are complex to objectify) that algorithms have difficulty considering, although they are at the core of clinical practice in psychiatry. Objectivity is a cultural and historical construct that has elicited different approaches over time and across cultures [125]. There has been a tendency to want to quantify illnesses ever since Claude Bernard introduced the nominalist approach denounced by Canguilhem [92]. Some see self-monitoring as the instrumentalization of health, promoting the proliferation of digital tracking tools with spurious ethical claims [126]. Even more so than other specialties, psychiatry may see itself as alienated from so-called algorithmic governmentality and, paradoxically, adopt the fantasy of illness-centered medicine curing psychiatric illnesses. Algorithmic governmentality does away with direct human interaction. Rouvroy and Stiegler [112] and Rouvroy and Berns [127] introduced this notion, defining it as derivative, with norm production reliant on massive data sets and regulations favored over humans’ anticipatory ability. With DP, psychiatry could therefore end up being driven by unreflective and determined rules instead of by the desire to create greater freedom to achieve goals. The risk is that it depends less on the HCP’s experience and allows less space for patients to express themselves [128]. DP may also lead us to a hostile world, driven by financial interests, where mental health becomes an unregulated market [129]. In addition, there is a risk of psychiatry becoming purely defensive [130], where medical tests or coercion are used under legal constraints. Finally, although DP represents an opportunity for patients to express themselves through technologies, it may bring more anxiogenic modalities of communication and hinder relationships [113], whereas they are actually crucial for patient mental health care.

### Alienation Instead of Empowerment

**Figure 2:**
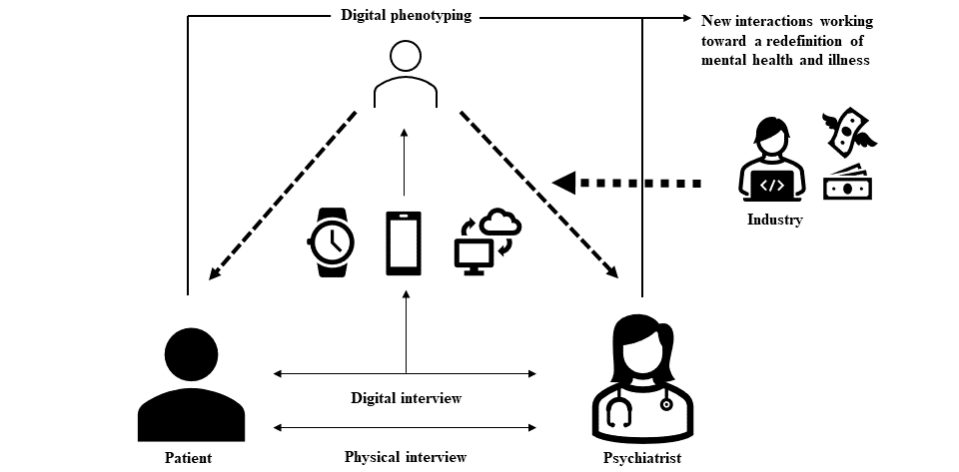
Conceptual illustration of the links created by the introduction of digital phenotyping into the health care relationship.

The opportunity for social and human progress in patients’ empowerment in psychiatry remains unclear. DP represents an instrument of biopower (derived from Foucault’s biopower [131,132]) for users, independently of its efficiency. The growing consideration of digital data in decision-making risks dispossessing humans (HCPs and patients) of the tools for producing health data. It may create a dependence on production tools, as already observed in the world of scientific research. Stiegler [133] demonstrated how researchers’ daily lives have been changed by the arrival of more and more digital interfaces between the objects they observe and the data required to produce knowledge. Stiegler [133] claimed that scientists are thus deprived of their production as they have to pay private industry for the right to use their own discoveries. DP could easily spark a similar process in the mental health industry [134], where the production of feelings becomes a patentable technique, introducing a third party in the relationship established by the clinical interview. Finally, DP risks defining mental health goals and dictating the means of attaining them (Figure 2). Illnesses may become political, part of a biopower seeking social control [135-137]. The term mental disorder used in the DSM-5 (instead of illness or disease) speaks for itself, the implicit meaning being that good mental health corresponds to a supposed mental order: illnesses render individuals unfit and remove their autonomy, such that they are dependent on the quantified self or on medical authorities (specialists) with increasingly narrow areas of expertise. Indeed, the current trend in psychiatry is to develop expert centers in which patients undergo a single clinical assessment, resulting in fast diagnosis and therapeutic guidelines. This paternalistic psychiatric assessment has little to do with the patient’s own expertise, which only HCPs who provide follow-up and long-term care can fully know. The risk is that HCPs relying on this data set will reach their diagnosis too early, interfering in the treatment procedure and compromising the follow-up needed to cure psychiatric illnesses.

### Ethical Issues

The concerns of patients whose intrinsic ability to normalize their environment is impaired are regarded as lying on the border between the normal and the pathological. DP could shift these borders by entering the patient-HCP relationship with a third party (Figure 2) or a fourth party if the relationship includes a family member or trusted person [138]. It raises the issue of responsibility, which also concerns the DP designer, manager, or analyst. Technological tools are not autonomous. DP threatens to go against the principles governing new technologies, namely neutrality, diversity, transparency, equity, loyalty, and overall comprehensibility.

Equity has been identified as one of the main ethical challenges of digital health [139]. Indeed, algorithms are constructed on complex models with user interfaces that are often difficult to understand. If users are not trained, DP will be misused due to the learning bias. HCPs and users could be victims of the black box effect, which can hinder the traceability of the decision-making process. It may well exacerbate the existing inequality of access to psychiatric care, penalizing patients from underprivileged backgrounds [94], on the wrong side of the digital divide [140], or exposed to the stigma [141] that extensively affects the psychiatric population [142]. Studies on the use of health data also tend to show that they are more acceptable to younger psychiatric patients [143], and adherence is predicted by a higher education level in schizophrenia [144]. There is therefore a risk that these technological advances will only benefit individuals who already enjoy easier access to psychiatric care.

In psychiatry, respect for privacy and confidentiality is also an essential part of the medical principle of patient autonomy, but health digitalization has brought about a paradigm shift. The sharing of information between different HCPs is now considered beneficial for patients. Professional integrity is replacing confidentiality [145,146]. Data anonymization can easily be bypassed by cross-referencing. Thus, personal health threatens to move from the private to the public arena [139] or to be extensively traded by private companies, particularly in psychiatry, where intimate data are of utmost importance for clinical practice. DP has indeed the potential for intrusion that would go against medical confidentiality, with questioning about the patients’ consent to share their health data. Hence, informed consent and data ownership are among the main issues [95,139,147].

Phenomenology currently lies at the core of mental health but risks being dislodged. Good health may contain a moral imperative, with pathologies being attributed either to alienation from the environment (nurture) or to biological predispositions (nature). When patients are freed of all responsibility in this way, failure ceases to exist and is replaced by illnesses, as has already happened with learning disorders, substance use disorders, and other deviances, ultimately leading to uneasiness [148]. Indeed, DP could stimulate nudging strategies, defined as “any aspect of the choice architecture that alters people’s behavior in a predictable way without forbidding any options or significantly changing their economic incentives” [149]. Nudges could create artificial health but also artificial illnesses, compromising patients’ autonomy [150,151] and agency [152]. Thus, it could be a barrier to meeting their mental health needs while creating new ones. The drift toward normativity through the medicalization of society can already be observed with the cult of performance [153] and the illusion of omnipotence over illness and death. Based on the notion of personal development, the post-Freudian therapies that first emerged back in the 1960s are intended to enable the immediate gratification of drives, thus increasing the isolation of the self [154]. The ultimate goal is for individuals to fulfill their personal achievement goals by worshiping authenticity and assertiveness, thereby exacerbating the very symptoms these therapies claim to cure. By further increasing the emphasis on performance, DP could contribute to the loss of identification with generational continuity, in other words, the inability of individuals to gradually identify themselves with the wellness and success of others rather than their own and, with the fear of growing old, the inability to think about their posterity and the handing of the baton to the next generation. The COVID-19 pandemic may be a good example of the counterhistorical health trade-off between generations: the sacrifice of liberty on behalf of health, with the collateral damage of poorer mental health among young people [155,156].

More and more digital mental health apps are available in web-based sales spaces. However, their design is seldom inspired by rigorous scientific research [105]. The popularity of these apps shows that there is consumer demand. The same need manifests itself when patients turn to complementary medicine because their physician fails to provide satisfaction. We can assume that through their use of these apps, individuals are not only responding to various marketing ploys but are also seeking to improve their daily lives. The COVID-19 pandemic undoubtedly fostered this change in the relationship between individuals and connected health, but there is a dearth of qualitative research on the place that connected objects now have in people’s daily lives [118].

## Recommendations for Implementing Digital Phenotyping in Psychiatry

### Overview

DP gives psychiatry an opportunity to adopt more modern ways of helping patients. However, it raises crucial questions about the values underpinning the definition of mental health [157] and could profoundly undermine the “sacred trust” that patients place in their physician to understand their illness. All in all, interdisciplinary collaboration in DP research is necessary [25,158], fostering expertise in psychiatry, computer science, data science, innovation, ethics, law, and the social sciences, to ensure the development of robust and clinically meaningful DP tools [159]. Further recommendations are needed to ensure that there is a true revolution in mental health.

### Empowerment of Patients and Health Care Professionals

#### Usability

Progress must be made in this area to ensure adequate usability. This poses a considerable challenge owing to the properties of the data (ie, high volume, heterogeneity, noise, and sparseness) [33,108]. The Beiwe platform is an example of the will to gather and rationalize passive smartphone data to phenotype psychiatric illnesses [2]. Patients must be able to choose which DP tool they want to use, which data can be collected, for what purpose, and have the right to withdraw [139].

#### Empathy of Care

DP could contribute to the empowerment of HCPs if the main objective is to facilitate their operational and administrative tasks and enable them to make clinical decisions. At the very least, clinical decision support systems must always be subject to professional validation, and all the data used by the algorithm must be reported. AI-based data analysis should reduce the workload of HCPs and allow them to focus on the Hippocratic aspects of care, such that their relationships with their patients are more humanistic, empathic, and centered on their individuality in terms of history, daily life, and symptoms, thereby creating more room for psychotherapeutic approaches. It goes without saying that this can only happen if the number of HCPs is not reduced by public health policies.

#### Cooperation Between Patients and Health Care Professionals

Just as DP gives individuals new tools for understanding themselves and their behaviors, it opens up new prospects for sharing a common and practical definition of mental health. This definition should be shared, personalized, and evolutive. The objective of DP should be to foster better cooperation among HCPs, patients, and their environment and to enhance understanding of their interplay.

#### Maintaining a Critical Mindset

Information on treatments introduces cognitive bias during the decision-making phase [83]. DP should be used as a debiasing strategy and not simply to create yet another layer of information for HCPs. Patients and HCPs should nevertheless consider the risks associated with nudging with these technologies. DP only constitutes one of the data sets representing patients’ mental health, which should instead be about sensitive, pragmatic, and rational reasoning between patients and HCPs over time.

### Information

#### For Patients

To counter the black box effect and the dispossession of health data, some authors recommend the use of explainable AI to set out the reasoning behind the conclusions [160]. This would remove the potential obstacle to patients’ and HCPs’ empowerment posed by algorithms. However, few AI models are currently available [160], but they promise to encompass ethics, security, and safety concerns [161]. In all cases, information and education need to be provided to help users understand DP, how it works, its limitations, and its potential failures.

#### For Health Care Providers

Data education must also be provided to HCPs. In France, medical students are not yet taught about the use of digital devices to improve patient health. Furthermore, we know that psychiatry residents mistrust digital culture [162]. This could be a major strategic error, as new generations will have to work in a global and competitive world of medicine.

#### For Designers

Finally, education must also be provided to the people who design and analyze DP applications. There is a lack of standards for building these tools, and their design needs to be in accordance with patients’ and HCPs’ experiences.

### Vigilance

#### Security

Progress must be made regarding the security of the data collected [139], otherwise this technology will never be acceptable [138]. This is a particular priority in psychiatry, where it is a prerequisite for using data science. It is very much an ethical challenge, for if we want this technology to uphold the fundamental principles of privacy, transparency, informed consent, accountability, and fairness [163], the security issue must be resolved within the next few years [147].

#### Equity

It is essential for DP to be available to the whole psychiatric population. The MindLogger platform for mobile mental health assessment is an example of such an initiative to democratize the development of mental health apps [164].

#### Data Rationalization and Research Priorities

Large-scale longitudinal data sets with standardized evaluation metrics are needed to assess the potential impact of these technologies. The costs of developing such tools need to be set against the proven and expected benefits. Studies are needed to analyze the qualitative and quantitative impact of an electronic health society on patients [165,166] and must ensure that the definition of mental health is not based on an artificial boundary between the normal and the pathological and does not become distanced from the patient’s experience. Ethical aspects of digital health research need to be considered in every study: equity, replicability, privacy, and efficacy [167]. In particular, qualitative studies must be conducted to define the tools’ contents (content validity) before performing studies to validate these tools statistically and psychometrically (structural validity, internal validity, cross-cultural validity, measurement error and reliability, criterion validity) [93]. Transdisciplinary approaches, including phenomenology, must be adopted during this construction process so as not to lose sight of patient-reported outcomes. Concerning algorithms, machine learning, natural language processing, and expert systems are the most studied interventions [115]. Up until now, studies have focused on diagnosis, prognosis, risk management, or follow-up, have had strong biases, have not taken health care end users into account, and finally have not responded to needs, as shown by the concerns about low engagement [143].

#### Auditability

An independent committee is needed to provide a legal framework for the marketing of DP tools based on recommendations for their construction, validation, consideration of users’ feedback, ethical considerations, and costs for society. Thus, DP could follow the established norms of quality and safety and finally be cost-effective and feasible [168].

## Conclusions

At the turn of the 17th century, there was a move from prescientific and Hippocratic medicine to Promethean medicine as a result of scientific discoveries and technological advances in biology. The 20th century’s paradigm consisted of using evidence-based medicine to bring about pragmatic progress. Medical practice continues to evolve; patients are once again regarded as experts in their own symptoms, and their preferences are given the same importance as external clinical data and medical experience. DP paves the way for a redefinition of mental health, making it more subjective while taking advantage of 21st century technologies for preventive, predictive, effective, and personalized medicine. We noted a certain enthusiasm for these new technologies among the general public, but HCPs remain skeptical, wondering whether the progress touted by private companies is really relevant for all patients and all HCPs. There now needs to be a qualitative study comparing patients’ and HCPs’ perspectives on the implementation of DP in psychiatry. DP calls into question the validity of the risk-benefit ratio, bringing another way of expressing and understanding illnesses. Thus, the challenge of DP will be to let patients access their own state of health, creating a new dimension of care where the borders of mental health are extended and not constrained by more digital interfaces and their pitfalls. Algorithmic governmentality should not be used to decide whether or not individuals deserve mental health care. To conclude, the priority should be to improve the abilities of patients to deal with their difficulties. DP has its place in psychiatry, fostering patients’ empowerment in terms of their illnesses, their health, their own lives, and those of others.

## Abbreviations

AI: artificial intelligence
DP: digital phenotyping
DSM-5: Diagnostic and Statistical Manual of Mental Disorders, Fifth Edition
HCP: health care professional
RDoC: research domain criteria

## Data Availability

Data sharing is not applicable to this article as no data sets were generated or analyzed during this study.
